# Twenty Years of Lyme Borreliosis in the Netherlands: Temporal Trends in Seroprevalence and Risk Factors

**DOI:** 10.3390/microorganisms12112185

**Published:** 2024-10-30

**Authors:** B. J. A. Hoeve-Bakker, Oda E. van den Berg, H. S. Doppenberg, Fiona R. M. van der Klis, Cees C. van den Wijngaard, Jan A. J. W. Kluytmans, Steven F. T. Thijsen, Karen Kerkhof

**Affiliations:** 1 Centre for Infectious Disease Control, National Institute for Public Health and the Environment (RIVM), 3721 MA Bilthoven, The Netherlands; dieneke.hoeve@rivm.nl (B.J.A.H.-B.); 2 Department of Medical Microbiology and Immunology, Diakonessenhuis Hospital, 3552 KE Utrecht, The Netherlands; 3 Department of Medical Microbiology, University Medical Center Utrecht, Utrecht University, 3584 CX Utrecht, The Netherlands; 4 Department of Medical Microbiology and Infectious Diseases, Erasmus University Medical Center, 3015 GD Rotterdam, The Netherlands

**Keywords:** *Borrelia*, C6 Lyme ELISA, epidemiology, immunoblot, multivariable analysis, serology, standard two-tier testing, surveillance, tick-borne disease

## Abstract

Lyme borreliosis (LB) is not notifiable in many European countries, and the patchwork of surveillance strategies in Europe perpetuates knowledge gaps. In the Netherlands, LB incidence has been estimated from recurring general practitioner surveys since the 1990s. To complement the incidence data, this study aimed to estimate the prevalence of antibodies against *Borrelia burgdorferi* sensu lato in the general population of the Netherlands in 1995/1996, identify risk factors for seropositivity, and compare these findings to data from 2016/2017 to identify temporal trends. Sera from participants (n = 8041, aged 0–80 years) in a cross-sectional nationwide surveillance study were assessed for the presence of antibodies against *B. burgdorferi* s.l., using a screening ELISA and immunoblot confirmation. Risk factors associated with seropositivity were evaluated using multivariable analysis. A significant difference in weighted seroprevalence was observed between 1995/1996 (2.8%) and 2016/2017 (4.3%). In both cohorts, the seroprevalence was significantly higher among men than among women, and increased with age and tick bite frequency. The upward trend in age-specific seropositivity in individuals over 50 was steeper in 2016/2017 than in 1995/1996, possibly due to improved fitness among contemporary elderly, allowing increased outdoor activities. This study highlights significant trends in the seroprevalence of *B. burgdorferi* s.l. antibodies in the general population of the Netherlands over 20 years. The doubling of seroprevalence underscores the increasing burden of LB, and the importance of continued surveillance. Targeted interventions, particularly for elderly populations, may help raise awareness to the risks of tick bites and reduce the growing disease burden and societal costs associated with LB.

## 1. Introduction

Lyme borreliosis (LB) is the most prevalent tick-borne disease in the northern hemisphere and is caused by spirochetes of the *Borrelia burgdorferi* sensu lato complex. In North America, LB is primarily caused by *B. burgdorferi* sensu stricto, with erythema migrans (EM) and Lyme arthritis being the most frequently observed manifestations. In Europe and Asia, *B. afzelii*, *B. garinii*, and *B. burgdorferi* s.s. are the most prevalent causative agents of skin manifestations, Lyme neuroborreliosis and Lyme arthritis, respectively [1,2]. Other *Borrelia* species that have been identified as human pathogens include *B. mayonii* in North America and *B. bavariensis*, *B. spielmanii*, and *B. lusitaniae* in Europe, although only sporadic cases have been reported [3].

The LB incidence rates across Europe show a heterogeneous distribution with great variations observed at both the national and regional level [4]. However, comparisons are challenging due to the lack of standardization in surveillance methods and notification policies [5,6]. Nonetheless, there is a global increase in LB incidence, mainly attributed to the climate change-induced expansion of the tick habitat, although the percentage of *Borrelia*-infected ticks remains stable [4,7]. Also, changes in human behavior and landscaping bring humans and ticks closer together, resulting in an increased risk of LB infection. In addition, increased awareness among physicians and the general public plays an important role in the increased detection of cases [4,8].

In the Netherlands, the general practitioner (GP)-reported EM incidence has increased from 39 EM diagnoses per 100,000 inhabitants in 1994 to 134 in 2009 [8]. The upward trend was largely explained by the aforementioned factors; however, data on which factor contributed and to what extent are largely lacking. From 2010 onwards, the number of EM diagnoses stabilized, possibly due to successful public health education strategies, including “Week van de Teek” (a national awareness week with extensive media attention at the beginning of the tick season) and “Tekenradar” (a citizen science-supported dashboard for monitoring tick activity on a regional level) [9,10]. Monitoring the EM incidence through retrospective cross-sectional surveys among GPs is relatively straightforward and provides a good representation of the general population in the Netherlands.

Serosurveillance (i.e., determining prevalence of *B. burgdorferi* s.l.-specific antibodies in a representative population) complements the surveillance of clinical LB incidence as this strategy is unaffected by the level of awareness to tick bites and clinical manifestations. Therefore, besides clinical infections, serosurveillance also includes subclinical and missed infections. Moreover, the additional epidemiological data obtained through serosurveys may also provide insights into risk factors and spatiotemporal patterns. However, the true exposure may still be underestimated in seroprevalence data due to a lack of seroconversion or antibody decay over time, especially in symptomatic infections in which the decay is accelerated by antibiotic treatment [11]. Despite these limitations, trends in seroprevalence can still identify increases in exposure, thereby enhancing the effectiveness of incidence surveillance. This improvement supports public health education, professional training, and targeted prevention efforts, such as reducing host populations or tick numbers.

In the Netherlands, population-based biobanks have been established every decade since 1995 to evaluate the effectiveness of the Dutch national immunization program (the PIENTER project) [12,13,14]. Furthermore, these biobanks have been made available for other public health related research. Recently, the third and most recent biobank (PIENTER-3) was used to determine the seroprevalence to *B. burgdorferi* s.l. in the general population in 2016/2017 and to identify risk factors for seropositivity [15]. Building on this research, the current study used the first biobank (PIENTER-1) to estimate the seroprevalence to *B. burgdorferi* s.l. in the general population of the Netherlands and to identify risk factors for seropositivity in 1995/1996, a period with less awareness of tick-borne diseases. In addition, the results were compared to those obtained using the PIENTER-3 biobank to identify trends on population-level *B. burgdorferi* s.l. infection over time. These trends may aid to improve public health interventions to reduce the burden of LB.

## 2. Materials and Methods

### 2.1. Study Population

In the Netherlands, the first national biobank for population-based cross-sectional seroprevalence studies (PIENTER-1) was established in 1995/1996 [14]. The biobank includes a national sample drawn from eight randomly selected municipalities in each of five geographic regions in the Netherlands. From each of these 40 municipalities, an age-stratified sample was drawn from the population register. To gain insight into the effects of clusters of unvaccinated individuals on herd immunity, a separate sample was drawn from eight municipalities with low vaccination coverage (LVC) in the national immunization program. The PIENTER-1 biobank contained 9948 serum samples and questionnaires from participants in the national sample (n = 8359) and the LVC (n = 1589).

The third national biobank (PIENTER-3) was established in 2016/2017 using a similar design as PIENTER-1, but was sampled independently [12]. The overlap in selected municipalities was therefore coincidental. The seroprevalence of *B. burgdorferi* s.l. antibodies in the general population and risk factors for seropositivity were previously described using the PIENTER-3 biobank [15]. For optimal comparison with the PIENTER-1 cohort, the oversampling group of non-Western migrants living in the Netherlands and participants older than 80 years were excluded from further analysis in the current study. The remaining PIENTER-3 data set included 5043 participants, of whom 4036 were in the national sample and 1006 were in the LVC (Supplementary Figure S1).

### 2.2. Laboratory Analysis

After retrieval from the biobank facility, the sera were stored and kept at −20 °C until testing. Before testing, the sera were thawed to room temperature, homogenized, and centrifuged.

**Figure 1:**
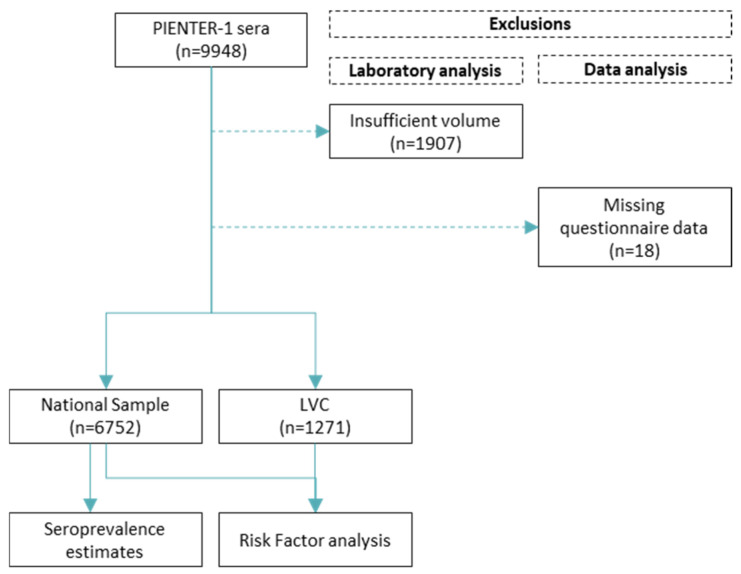
Schematic representation of the study design for the PIENTER-1 cohort. LVC, low vaccination coverage sample.

Of the 9948 sera in the PIENTER-1 biobank, 1907 were excluded due to insufficient volume (<100 µL) (Figure 1). The remaining 8041 sera were tested for immunoglobulin (Ig) M and IgG antibodies against *B. burgdorferi* s.l. using standard two-tier testing as described previously [15]. In short, sera were screened with the C6 Lyme ELISA (Immunetics, Boston, MA, USA), and sera with a negative result were considered seronegative. Equivocal and positive screening results were retested with the recomLine Borrelia IgM and IgG immunoblots (Mikrogen, Neuried, Germany). Negative IgM and IgG results and solitary equivocal IgM results were considered seronegative. Solitary equivocal IgG results and positive IgM and/or IgG results were considered seropositive.

### 2.3. Data Analysis

Upon addition of the questionnaire data to the laboratory results of the PIENTER-1 participants, 18 participants were excluded due to absent questionnaire data (Figure 1). Of the included participants, 6752 were in the national sample and 1271 were in the LVC.

Data analyses regarding demographics, seroprevalence estimates and risk determinants were performed as described previously [15]. To estimate the seroprevalence for *B. burgdorferi* s.l.-specific antibodies from the national sample, seroprevalence was weighted within each municipality for age, sex, ethnic background, and degree of urbanization proportional to the distribution of the general population in the Netherlands. The analyses were further adjusted for the survey design by taking into account the strata (geographic regions) and clusters (municipalities). Risk factor analysis was performed to identify variables associated with *B. burgdorferi* s.l. seropositivity. To increase power, data from the LVC were also included, as the sample group was not associated with *B. burgdorferi* s.l. seropositivity. Univariable analysis using logistic regression was carried out on selected explanatory questionnaire variables against binary serology results (positive or negative), with a priori adjustment for age and sex. Explanatory variables that reached a significance level of *p*-value < 0.10 were included in the multivariable logistic regression model. Multicollinearity was tested using the variance inflation factor, and variables with a VIF > 4 were excluded from the model. The multivariable model was optimized further using stepwise backward elimination based on Akaike’s Information Criterion (AIC).

Data management and analyses were conducted in R version 4.3.2 [16].

## 3. Results

### 3.1. Study Population

Of the 6752 participants included in the national sample of PIENTER-1, 46.7% were men and 53.3% women (Table 1). The youngest age group (0–19 years) was slightly over-represented, but participants were equally distributed regarding geographic region and socioeconomic status (SES). The majority were autochthonous to the Netherlands (88.8%), and reported no tick bites in the last 5 years (90.4%).

**Figure 2:**
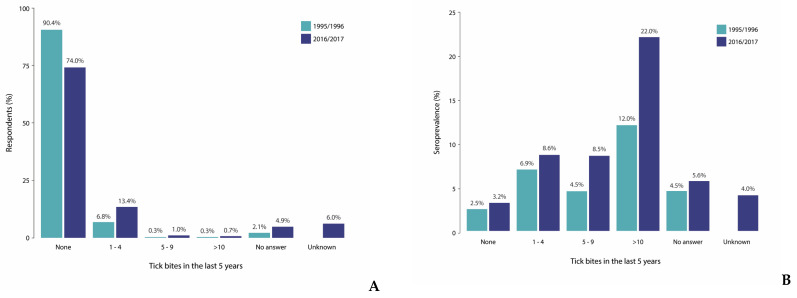
Self-reported tick bites in the last 5 years in 1995/1996 (green) and 2016/2017 (purple). (**A**) Tick bites reported by the participants; (**B**) Seroprevalence per reported tick bite frequency.

The national sample of the PIENTER-3 cohort was distributed comparably to the PIENTER-1 national sample with regard to sex, ethnicity, and SES (Table 1). However, the proportions of the age groups 0–19 years and 20–39 years were lower and higher, respectively. Also, the central and eastern regions of the Netherlands were slightly over-represented at the expense of the western regions. In PIENTER-3, fewer participants reported no tick bites, whereas the proportions of participants that did report tick bites at least doubled in all three frequency groups (Table 1, Figure 2A). In contrast to PIENTER-1, the PIENTER-3 questionnaire included an option for participants to indicate if they did not know whether they had been bitten, and 6.0% of the participants indicated this.

### 3.2. Laboratory Analysis of the PIENTER-1 Sera

The PIENTER-1 sera were screened for *B. burgdorferi* s.l.-specific IgM and IgG antibodies using the C6 Lyme ELISA. The result was negative for 7171 (89.2%) of the 8041 tested sera. The 870 (10.8%) reactive sera were retested using an immunoblot. Of these, 640 (73.6%) were negative for both IgM and IgG, and 1 (1.1%) serum was equivocal for IgM only. Of the remaining 229 (26.3%) sera that were reactive in the immunoblot, 30 (3.4%) were positive for IgM only, 47 (5.4%) were equivocal for IgG only, and 116 (13.3%) were positive for IgG only. Furthermore, 2 (0.2%) sera were positive for IgM and equivocal for IgG, 5 (0.6%) equivocal for IgM and positive for IgG, and 27 (3.1%) were positive for both isotypes. Lastly, two sera (0.2%) could not be tested for IgM, but were positive for IgG (Supplementary Table S1).

### 3.3. Seroprevalence Estimates in 1995/1996

**Figure 3:**
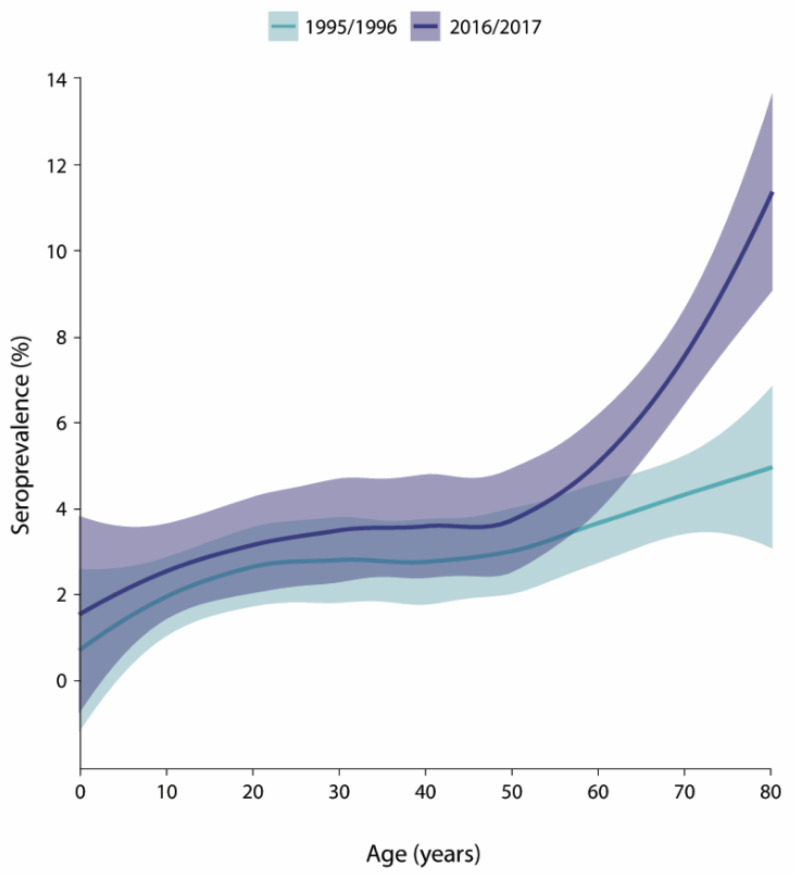
Smooth age-specific seroprevalence of *B. burgdorferi* s.l. antibodies in the general population of the Netherlands in 1995/1996 (green) and 2016/2017 (purple). Shaded areas indicate 95% confidence intervals.

**Figure 4:**
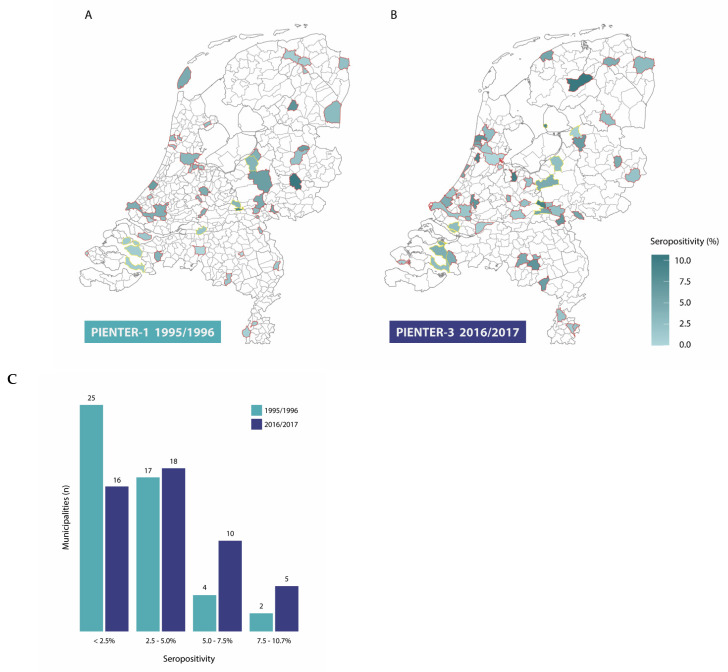
Lyme borreliosis seropositivity in the sampled municipalities in the Netherlands in 1995/1996 (**A**) and 2016/2017 (**B**). Red and yellow boundaries indicate municipalities in the national sample and low vaccination coverage sample, respectively. (**C**): number of municipalities stratified by seropositivity in 1995/1996 (green) and 2016/2017 (purple).

In 1995/1996, the weighted *B. burgdorferi* s.l. seroprevalence for individuals aged 0–80 was 2.8% (95% CI 2.3–3.4) (Table 1). The seroprevalence estimate was higher among men (3.6%) than among women (2.1%) and increased with age from 1.5% among 0- to 19-year-olds to 4.5% among 60- to 80-year-olds (Table 1, Figure 3). The seropositivity in the sampled municipalities ranged from 0.0% to 10.6%, although the seropositivity was below 5% for the majority of the municipalities (87.5%) (Figure 4A,C). The seroprevalence was highest in the central region (5.2%) of the Netherlands and considerably lower in the other four regions (1.3% to 3.2%) (Table 1). The seroprevalence increased with tick bite frequency from 2.5% among participants that reported no tick bites to 12.0% among those that reported >10 tick bites in the last five years (Table 1, Figure 2B).

### 3.4. Seroprevalence Estimates in 2016/2017 and Trend Analysis

In 2016/2017, the seroprevalence increased to 4.3% (95% CI 3.4–5.1), and this was significantly higher than 2 decades earlier (Pearson’s chi-squared test, *p*-value 0.009). As in 1995/1996, the seroprevalence was also higher among men than among women (5.4% respectively 3.1%) in 2016/2017. A similar increasing trend was observed regarding age, although the offset was higher (2.7% in age group 0–19 years) in 2016/2017. Between 0 and 20 years of age, an increase in the seroprevalence was observed in both cohorts, which stagnated until approximately 50 years of age (Table 1, Figure 3). The observed increase from 50 years onwards was, however, steeper in 2016/2017 than in 1995/1996. Although different municipalities were sampled in the PIENTER-3 study, the range of seropositivity percentages among municipalities was comparable to PIENTER-1 (0.0% to 10.7%) (Figure 4B). In 2016/2017, however, the number of municipalities with seropositivity >5% had increased compared to 1995/1996 (Figure 4C). Contrary to 1995/1996, the seroprevalence in 2016/2017 was more consistently high (3.0 to 5.0%) in all five regions of the Netherlands (Table 1). The seroprevalence in relation to tick bite frequency showed a similar upward trend as in 1995/1996, increasing from 3.2% among participants that reported no tick bites to 22.0% among those reporting more than ten tick bites (Table 1, Figure 2B).

### 3.5. Risk Determinants

In the PIENTER-1 cohort, men had a 1.63 (95% CI 1.24–2.13) times higher odds of being seropositive than women (Table 2). The odds of being seropositive also increased with age, and were significantly higher for age groups 40–59 (aOR: 2.06, 95% CI 1.37–3.08) and 60–80 (aOR: 2.85, 95% CI 1.92–4.25). The participants living in the central region of the Netherlands had a 3.32 (95% CI 1.82–6.03) times higher odds of being seropositive than those living in the southeast. The odds of being seropositive also increased with the number of reported tick bites, up to an aOR of 3.7 (95% CI 1.08–12.72) for those reporting more than ten tick bites. However, for those reporting five to nine tick bites, the odds did not significantly differ from the reference group. Lastly, having a rheumatic disorder (aOR: 1.89, 95% CI 1.04–3.45) and keeping pigs (aOR: 4.42, 95% CI 2.16–9.06) were identified as risk determinants for seropositivity (Table 2).

Two decades later, the odds of being seropositive remained similarly higher for men (aOR: 1.62, 95% CI 1.22–2.14) in the PIENTER-3 cohort (Table 2). Although the increasing trend with age remained, the odds were only significantly higher for the participants in the highest age group (aOR: 3.58, 95% CI 2.27–5.64). The trend of the odds increasing with tick bite frequency was more pronounced than in PIENTER-1, with almost tenfold increased odds of being seropositive for participants reporting ten or more tick bites (aOR: 9.77, 95% CI 4.37–21.85). In contrast with the PIENTER-1 cohort, the geographic region of residence, having a rheumatic disorder, and keeping pigs were no longer identified as risk determinants for seropositivity.

## 4. Discussion

In this study, the seroprevalence for *B. burgdorferi* s.l. antibodies was estimated for the general population of the Netherlands in 1995/1996, and the risk factors for seropositivity were assessed. Furthermore, the results from this cohort were compared with a similar cohort that was established 20 years later when public education practices were in place. The observed trends together with incidence-based surveillance data can help assessing the trends and the impact of LB and other tick-borne diseases.

In 1995/1996, the *B. burgdorferi* s.l. seroprevalence in the general population of the Netherlands was 2.8%. The observed seroprevalence was similar to percentages reported among blood donors in other contemporary studies in Western Europe that ranged from 2% to 8% [17]. In the Netherlands in 1989, higher seroprevalence percentages were observed among healthy blood donors (8.7%) and male office personnel (5%) [17]. Those seroprevalence percentages might, however, be an overestimation, since an in-house screening ELISA with a whole-cell lysate antigen was used instead of the recommended two-tiered testing strategy applied in the current study. Moreover, Nohlmans et al. reported a high interlaboratory variability of their in-house assay, which might suggest suboptimal test performance [17].

Two decades later, in 2016/2017, the *B. burgdorferi* s.l. seroprevalence in the general population of the Netherlands had increased significantly to 4.3%. Insights into temporal dynamics of seroprevalence are scarce and the reported temporal trends across Europe are diverse. A similar increasing trend was reported in Germany, where the seroprevalence slightly increased from 8.5% in 1997–1999 to 9.3% in 2008–2011 [18]. Contrastingly, the reported seroprevalence in Finland was 20.0% in 1968–1972, which decreased to 3.9% in 2011 [19,20]. High exposure to ticks during work and outdoor recreational activities in the agrarian society in Finland was postulated to have attributed to the high seroprevalence in the 60s/70s [20].

Overall, the *B. burgdorferi* s.l. seroprevalence in the Netherlands has almost doubled in two decades, which demonstrates that at least twice as many people had actually been infected. The significant increase in total surface of suitable tick habitats in the Netherlands, and the rising annual number of warm days favoring prolonged tick activity, provide evidence for a rise in the abundance of *B. burgdorferi* s.l.-infected ticks [21]. Moreover, human exposure might have increased due to the climate change-induced expansion of tick habitats into urban green spaces and altered human risk behavior (i.e., increased outdoor activity) [7].

The seroprevalence did, however, not increase proportionally to the previously reported a fourfold increase in GP-reported EM diagnoses and an almost threefold rise in reported tick bites (615,000 in 1995/1996 to 1.5 million in 2016/2017) [8,9,22]. This lagging increase in seroprevalence may be explained by increased awareness of ticks due to public health education strategies such as “Week van de Teek” and “Tekenradar”, leading to earlier tick removal and thus reduced transmission risk [23]. Moreover, increased awareness has led to more EM diagnoses, and the prescribed antibiotic treatment may have prevented seroconversion and/or accelerated antibody decay [8,10,11].

Risk factors for seropositivity in both PIENTER cohorts included being male, increasing age, and increasing tick bite frequency. Increasing tick bite frequency as a risk factor for seropositivity is a logical biological association that was demonstrated in several studies involving study populations with increased tick exposure [24,25,26]. However, the present study and our previous studies are the first to demonstrate this association in the general population [15]. The tick bite frequency in the last five years in the PIENTER cohorts is, however, self-reported and is therefore prone to recall and telescoping bias, which might have led to an overestimation of the number of tick bites per participant. On the other hand, some tick bites were inevitably missed, as evidenced by the observed seropositivity among those that reported no tick bites in the last five years. The PIENTER-3 questionnaire aimed to mitigate the effect by offering the additional option “I don’t know”, and the seropositivity in this group was slightly higher than among participants that reported no tick bites in the past five years. Another option would be a sensibility analysis, asking the same questing over a shorter interval, where recall and telescoping bias is expected to be less significant.

The observed higher seroprevalence among men did not change over time and supports the many previous observations of increased risk for seropositivity among men [18,19,25]. Similar trends were also demonstrated in populations with increased exposure, i.e., forestry workers, suggesting other underlying causes besides exposure time. For example, women demonstrate more proactive health care seeking behavior, which may result in earlier diagnosis and proper treatment of LB. This is supported by higher disease incidence of early localized manifestations among women, while disseminated LB is more frequently observed among men [27,28].

Increasing age as a risk factor for seropositivity is the result of increasing cumulative exposure time. Other proposed explanations, however, might include increased outdoor activity among the elderly, boosting the effect of reinfection or the lack of antibody clearance [19]. Until 50 years of age, the age-stratified seroprevalence increased evenly between both PIENTER cohorts. Among 50- to 80-year-olds, however, the seroprevalence increased significantly more steeply in 2016/2017 than in 1995/1996. Increased fitness and outdoor activity among the elderly over time may have contributed to higher seropositivity percentages [29]. As the LB incidence generally increases with age, the observed trend might also result in more clinical infections in this age group, and should be studied further [30]. Furthermore, public health education tailored towards elderly could increase awareness among elderly and their physicians, and reduce disease burden and societal costs of LB.

Rheumatism and keeping pigs were associated with *B. burgdorferi* s.l. seropositivity in the PIENTER-1 cohort, but not in the PIENTER-3 cohort. Rheumatism is a plausible risk factor for seropositivity, since Lyme arthritis may manifest with complaints resembling rheumatism. While the proportion of participants reporting rheumatic complaints remained stable, as did the seropositivity percentage within this group, the significant increase in the overall seroprevalence rendered rheumatism no longer a risk factor in the PIENTER-3 cohort. The association between keeping pigs and *B. burgdorferi* s.l. seropositivity was no longer observed within the PIENTER-3 cohort, likely due the substantial decrease in the proportion of pig farmers.

The PIENTER project provided a unique and extensive data set, representative of the general population in the Netherlands. Both biobanks used in this study were compiled using the same strategy, which provided optimal conditions for the comparison and identification of temporal trends. Both biobanks were, however, sampled independently, and, consequently, all comparisons are population-based. During the COVID-19 pandemic, a subset of the PIENTER-3 participants was included in a follow-up study (PIENTER-Corona) [31], and this longitudinal data set could in future studies provide additional information regarding infection dynamics such as seroconversion and seroreversion rates.

The sera of the PIENTER-1 biobank had been stored for 25 years at the time of analysis, which might be considered a limitation. The sera were aliquoted to prevent repeated freeze-thawing that might decrease the quality of the sera and stored at −86 °C [32]. The stability of the PIENTER-1 biobank was confirmed by re-evaluating a random sample 10 years later, yielding consistent results (personal communication Titia Kortbeek, RIVM, the Netherlands), supporting the findings of Dard et al. [33]. Therefore, there was no reason to assume significant deterioration of *B. burgdorferi* s.l. antibodies in the PIENTER-1 biobank.

## 5. Conclusions

This study highlights important temporal trends in the seroprevalence of *B. burgdorferi* s.l. antibodies in the general population of the Netherlands over a span of two decades. The doubling of seroprevalence underscores the increasing burden LB in the region and emphasizes the importance of continued surveillance efforts in combating tick-borne illnesses. The observed associations between seropositivity and risk factors such as gender, age, and self-reported tick bite frequency provide valuable insights for public health strategies aimed at prevention and education. Moving forward, targeted interventions, particularly tailored towards elderly populations, may help mitigate the growing disease burden and societal costs associated with LB.

## Figures and Tables

**Table 1:** Sociodemographic characteristics of the participants and weighed seroprevalence of anti-*B. burgdorferi* s.l. antibodies in the general population of the Netherlands in 1995/1996 and 2016/2017.

	1995/1996						2016/2017				
	Total			Weighted Anti-*B. burgdorferi* s.l. Antibodies Seroprevalence			Total			Weighted Anti-*B. burgdorferi* s.l. Antibodies Seroprevalence	
	n	%		%	95% CI		n	%		%	95% CI
**Overall**	6752	100.0		2.8	2.3–3.4		4036	100.0		4.3	3.4–5.1
**Sex**											
Female	3598	53.3		2.1	1.5–2.8		2222	55.1		3.1	2.3–4.1
Male	3154	46.7		3.6	2.8–4.5		1814	44.9		5.4	4.3–6.8
**Age (years)**											
0–19	2082	30.8		1.5	1.1–2.1		750	18.6		2.7	1.4–4.6
20–39	1417	21.0		2.9	2.0–4.0		1307	32.4		3.4	2.3–5.0
40–59	1745	25.8		3.1	2.2–4.2		1068	26.5		3.8	2.5–5.4
60–80	1508	22.3		4.5	3.0–6.4		911	22.6		7.7	6.0–9.7
**Region ^1^**											
Northeast	1439	21.3		3.2	1.3–6.3		898	22.2		5.0	2.6–8.6
Southeast	1429	21.2		1.3	0.4–2.8		959	23.8		4.6	3.3–6.2
Central	1120	16.6		5.2	3.6–7.2		834	20.7		3.8	2.0–6.7
Southwest	1353	20.0		2.4	1.1–4.7		692	17.1		3.0	1.5–5.5
Northwest	1411	20.9		2.6	1.9–3.5		653	16.2		4.7	2.1–9
**Ethnicity**											
Dutch	5999	88.8		3.0	2.3–3.7		3530	87.5		4.5	3.6–5.5
Immigrant	753	11.2		2.3	1.4–3.6		506	12.5		3.7	2.1–5.8
**Socioeconomic status ^2^**											
low	2460	36.4		2.8	1.9–3.9		566	14.0		3.5	2.2–5.2
middle	2116	31.3		2.8	1.9–3.9		1364	33.8		3.1	2.2–4.2
high	2108	31.2		3.0	2.2–4.0		1869	46.3		5.8	4.3–7.5
**No. of tick bites in the last 5 years**											
Never	6106	90.4		2.5	1.9–3.1		2988	74.0		3.2	2.5–4.0
1–4 times	457	6.8		6.9	3.9–11.3		540	13.4		8.6	5.8–12.2
5–9 times	23	0.3		4.5	0.1–24.2		42	1.0		8.5	2.3–20.7
≥10 times	23	0.3		12.0	2.0–34.0		28	0.7		22.0	8.9–41.1
Unknown							242	6.0		4.0	2.0–7.1

CI, confidence interval. ^1^ Regions comprised the following provinces: Northeast: Groningen, Friesland, Drenthe, and Overijssel; Southeast: Noord-Brabant and Limburg; Central: Gelderland and Utrecht; Southwest: Zeeland and Zuid-Holland; Northwest: Noord-Holland and Flevoland. ^2^ Socioeconomic status was classified based on highest completed education of the participant. For children <18 years, the highest completed education of either parent was taken. Low: primary or lower vocational or general secondary education; middle: intermediate vocational or general secondary education, or higher general secondary education; high: higher vocational secondary or university education.

**Table 2:** Risk determinants for *B. burgdorferi* s.l. seropositivity in the PIENTER-1 and PIENTER-3 cohorts.

	1995/1996						2016/2017				
	n	%	n_pos_	%_pos_	aOR [95% CI]		n	%	n_pos_	%_pos_	aOR [95% CI]
**Sex**											
Female	4263	53.1	97	2.3	Reference		2824	56.0	93	3.3	Reference
Male	3760	46.9	131	3.5	1.63 [1.24−2.13]		2218	44.0	122	5.5	1.62 [1.22−2.14]
**Age (years)**											
0–19	2473	30.8	38	1.5	Reference		955	18.9	26	2.7	Reference
20–39	1682	21.0	40	2.4	1.52 [0.97−2.38]		1666	33.0	50	3.0	1.25 [0.77−2.04]
40–59	2074	25.9	69	3.3	2.06 [1.37−3.08]		1333	26.4	51	3.8	1.61 [0.99−2.61]
60–80	1794	22.4	81	4.5	2.85 [1.92−4.25]		1088	21.6	88	8.1	3.58 [2.27−5.64]
**No. of tick bites in the last 5 years**											
Never	7279	90.7	177	2.4	Reference		3735	74.1	119	3.2	Reference
1–4 tick bites	522	6.5	40	7.7	2.85 [1.97−4.13]		671	13.3	51	7.6	2.58 [1.83−3.65]
5–9 tick bites	23	0.3	1	4.3	0.86 [0.11−6.94]		51	1.0	5	9.8	3.50 [1.33−9.19]
≥10 tick bites	28	0.3	3	10.7	3.70 [1.08−12.72]		35	0.7	9	25.7	9.77 [4.37−21.85]
Unknown							309	6.1	17	5.5	1.62 [0.95−2.74]
**Region ^1^**											
Southeast	1578	19.7	25	1.6	Reference		959	19.0	40	4.2	n.s.
Central	1483	18.5	79	5.3	3.32 [1.82−6.03]		1250	24.8	49	3.9	
Northeast	1439	17.9	45	3.1	1.86 [0.97−3.57]		1033	20.5	48	4.6	
Northwest	1411	17.6	37	2.6	1.73 [0.89−3.35]		780	15.5	44	5.6	
Southwest	2112	26.3	42	2.0	1.33 [0.71−2.48]		1020	20.2	34	3.3	
**Rheumatism**											
No	7655	95.4	214	2.8	Reference		4255	84.4	193	4.5	n.s.
Yes	213	2.7	13	6.1	1.89 [1.04−3.45]		133	2.6	8	6.0	
**Keeping pigs**											
No	7791	97.1	211	2.7	Reference		5017	99.5	215	4.3	n.s.
Yes	86	1.1	10	11.6	4.42 [2.16−9.06]		22	0.4	0	0	

aOR, adjusted odds ratio. CI, confidence interval. ^1^ Regions comprised the following provinces: Northeast: Groningen, Friesland, Drenthe, and Overijssel; Southeast: Noord-Brabant and Limburg; Central: Gelderland and Utrecht; Southwest: Zeeland and Zuid-Holland; Northwest: Noord-Holland and Flevoland. Missing: no. of tick bites in the last 5 years, 171 (PIENTER-1), 241 (PIENTER-3); rheumatism, 155 (PIENTER-1), 640 (PIENTER-3); keeping pigs, 146 (PIENTER-1), 3 (PIENTER-3).

## Data Availability

The original contributions presented in the study are included in the article/Supplementary Materials, further inquiries can be directed to the corresponding author.

## Supplementary Materials

The following supporting information can be downloaded at https://www.mdpi.com/article/10.3390/microorganisms12112185/s1, Supplementary Figure S1. A flow chart of the study setup for the PIENTER-3 biobank in the current study. The seroprevalence of *B. burgdorferi* s.l. antibodies in the general population and risk factors for seropositivity were previously described using the PIENTER-3 biobank [10]. For optimal comparison with the PIENTER-1 cohort, the oversampling group of non-Western migrants living in the Netherlands, and participants older than 80 years were excluded from further analysis in the current study. The laboratory results of the remaining 5043 PIENTER-3 participants (4036 in the national sample and 1006 in the low vaccination coverage sample [LVC]) were used for analysis, Supplementary Table S1. Laboratory results of the PIENTER-1 biobank using the standard two-tier testing strategy. Sera were screened using the C6 Lyme ELISA. Positive or equivocal screening results were confirmed using the recomLine Borrelia IgM and IgG immuno-blots.
